# The XFEL Protein Crystallography: Developments and Perspectives

**DOI:** 10.3390/ijms20143421

**Published:** 2019-07-12

**Authors:** Haiguang Liu, Weontae Lee

**Affiliations:** 1 Complex Systems Division, Beijing Computational Science Research Center, Beijing 100193, China; 2 Physics Department, Beijing Normal University, 19 Xinjiekouwai St, Haidian, Beijing 100875, China; 3 Department of Biochemistry, College of Life Science & Biotechnology, Yonsei University, Seoul 03722, Korea

In the past 10 years, the world has witnessed the revolutionary development of X-ray free electron lasers (XFELs) and their applications in many scientific disciplinaries [1]. Lasers at wavelengths such as the visible light regime have been broadly applied in scientific research, industry, and even in daily life, mainly by utilizing the ‘bound’ electrons transiting from higher energy quantum states to lower states, but this mechanism limits the energy range of the emitted photons. The X-ray lasers became possible by passing high energy ‘free’ electrons through periodic alternating magnetic fields [2]. The commissioning of the Linac Coherent Light Source (LCLS) in 2009 marks a milestone for both laser and hard X-ray technologies [3]: the dream of X-ray lasers came true, and the constructions of several other multi-billion-dollar XFEL facilities immediately followed (see [4] and Figure 1 for the updated laser guns). XFELs produce fully coherent, ultrabright, femtosecond X-ray pulses (each containing about 1 × 10^11^–1 × 10^12^ photons), boosting the peak brilliance of X-ray sources by about 10 billion times compared to the third generation synchrotrons. The unprecedented bright X-ray beams have been applied to determine electron density distributions and electronic structures.

Thanks to the femtosecond X-ray laser pulses, experiments can be carried out in the so-called ‘diffraction-before-destruction’ mode (Figure 2): the X-ray beams are so bright that they destroy the samples after each exposure, yet the pulses are so short that the measurement (exposure) is finished before the onset of radiation damage [5,6,7,8]. Furthermore, experimental data have shown that the diffraction signals have a self-terminating nature when measured using XFEL pulses, i.e., the diffraction intensity would fade out as the crystalline orders are damaged by the X-ray radiation [9]. The X-ray wavelength is highly tunable at XFEL facilities, and even two-color X-rays can be generated by specially designed undulators [10]. These advancements make it possible to measure the anomalous diffraction signals for experimental phasing purposes [11] and the dynamic nature of protein at room temperature. One major application of XFEL protein crystallography is the structure determination of membrane proteins, which are naturally good targets for drug development. The atomic resolution structures of several important G-protein coupled receptor (GPCR) proteins have been resolved using the XFEL serial femtosecond crystallography (SFX) method [12,13]. Given the fast development of this emerging technology, four excellent articles related to XFEL protein crystallography are selected and published in this special issue.

There are four essential components in XFEL protein crystallography: (1) batched microcrystal sample preparation; (2) sample delivery and manipulation; (3) XFEL and instrument operation for diffraction data collection; and (4) data analysis and model interpretation. Four articles nicely cover components (1), (2) and (4). The sample delivery methods are summarized by Nam, with a focus on the sample delivery medium that carries the crystals to the XFEL interaction volume [14]. The sample preparations are well presented in the articles on XFEL applications in determining the structure and dynamics of the HIV integrase catalytic core domain (CCD) [15] and the HIV-1 Gag matrix domain with inositol hexaphosphate (MA-IP6) [16]. XFEL protein crystallography not only determines high resolution structures of proteins, but also reveals the time-stamped conformational changes of proteins. Schmidt reviews the time-resolved SFX using XFELs to study the ultrafast dynamics of protein molecules [17]. Data analysis has been a challenging task for time-resolved SFX because of the complexities raised in the pumping methods and the electron density re-arrangement upon pumping. The analysis protocol using extrapolated structure factors and model refinement provides a systematic approach to convert experimental data to dynamics information.

Compared to conventional crystallography, the SFX at XFELs has a few differences:

1. Each crystal usually results from a single diffraction pattern within tens of femtoseconds of exposure time, so the measured intensity for each reflection in a single pattern is incomplete (inaccurate) due to the excited volume defined by the overlapped region between the thin Ewald shell and the full reflection volume. Therefore, a high measurement redundancy is desirable for accurate diffraction intensity. This can be achieved by the high repetition rates of XFEL pulses. The processing of large data volumes requires special software for screening [18,19,20], auto-indexing [21,22,23], merging and also post-refinement, as summarized in [24].
2. With the ‘diffraction-before-destruction’ approach, the cryogenic protection of crystals is not required, so the experiments can be done with the samples at room temperature (if the temperature changes during the injection can be neglected). Therefore, the structures might be different from those determined at synchrotrons in some regions, as discussed by Park et al. in the HIV-CCD structure analysis [15]. Such comparisons may provide important clues for the understanding of protein functions and how they may apply to drug development [25].
3. With the femtosecond XFEL pulses, the temporal resolution can also reach the femtosecond time scale in theory, making it possible to study ultrafast dynamics. Light-triggered reactions are very suitable for time resolved SFX, revealing conformational changes down to a sub-picosecond time scale [26,27]. Enzymatic reactions or receptor conformational changes triggered by substrate binding are possible by using a fast mixing device or premixed solution of engineered substrates that are photocaged, although the time resolution will be limited to a longer time scale [28,29].
4. The ultimate goal of structure studies is to probe structure information from single molecules using XFELs, similar to the cryogenic electron microscopy method. Even with the most powerful XFELs, the resolution for single particle imaging can only reach a few nanometers for viruses [30,31]. Nonetheless, the ensemble measurement can yield interpretable solution scattering signals, providing dynamics information when combined with pump–probe technology [32,33].

The protein crystallography at XFELs has shown encouraging results—127 structures were found using XFEL as the searching keyword in the protein data bank when this manuscript was prepared. We would anticipate more structures reported using the XFEL crystallography method as several X-ray lasers are commissioned. Two super-XFELs, the LCLS-II in USA and the SHINE in China, each designed to produce up to 1 million XFEL pulses per second, are under construction. More excitingly, the time resolved crystallography at XFELs will capture the conformations of proteins in-action, producing three-dimensional (3D) molecular movies to reveal the functioning state down to atomic levels.

## Figures and Tables

**Figure 1:**
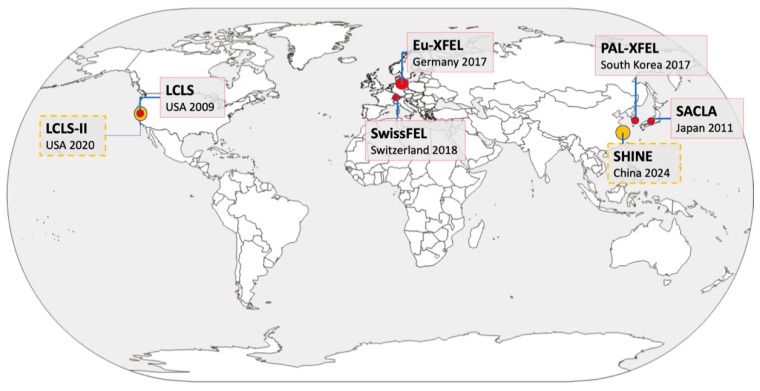
A map of X-ray free electron laser (XFEL) facilities. Five of these are in operation (red circles), and two facilities (Linac Coherent Light Source (LCLS)-II and Shanghai HIgh repetition rate XFEL aNd Extreme light facility (SHINE), orange circles) are designed to operate at 1 MHz in continuous wave (CW) mode, in contrast to the long pulse (LP) mode in Eu-XFEL.

**Figure 2:**
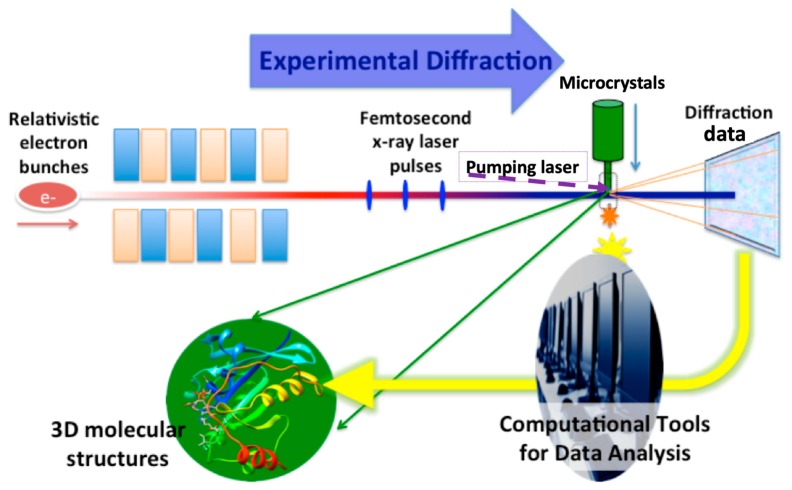
The workflow for protein crystallography at XFELs. The upper panel shows the forward process: high energy electrons produce coherent X-ray pulses that intercept microcrystals delivered via the injecting method, resulting in diffraction signals. The lower panel shows the data analysis and model interpretation, i.e., the inverse problem, to be solved using computational modeling methods. The pumping laser (purple dashed line) can be included for time-resolved experiments (Figure reproduced from [24] with permission).
